# Autonomic Nerve Activity Features According to Dry Eye Type

**DOI:** 10.1167/iovs.64.7.19

**Published:** 2023-06-12

**Authors:** Minako Kaido, Reiko Arita, Yasue Mitsukura, Kazuo Tsubota

**Affiliations:** 1 Wada Eye Clinic, Chiba, Japan; 2 Tsubota Laboratory, Inc., Tokyo, Japan; 3 Itoh Clinic, Saitama, Japan; 4 Faculty of Science and Technology, Keio University, Kanagawa, Japan

**Keywords:** autonomic nerve activity, coefficient of component variance (ccv), dry eye (DE), Japanese version of the Ocular Surface Disease Index (OSDI), parasympathetic nerve, stress

## Abstract

**Purpose:**

The purpose of this study was to investigate the association between autonomic nerve activity and symptom intensity according to the type of dry eye (DE).

**Methods:**

This prospective, cross-sectional, comparative study included 25 eyes of 25 patients with short tear break-up time DE (sBUTDE; mean age = 57.4 ± 11.4 years, range = 30–74 years) and 24 eyes of 24 patients with aqueous tear-deficient DE (ADDE; mean age = 62.3 ± 10.7 years, range = 29–76 years) were studied. Autonomic nerve activity was examined, and the Japanese version of the Ocular Surface Disease Index (J-OSDI) and a stress check questionnaire were administered. Autonomic nerve activity was continuously measured for 10 minutes. The parameters were low-frequency (LF) and high-frequency (HF) components of heart rate variability, reflecting cardiac sympathetic and parasympathetic nerve activity, and parasympathetic nerve activity alone, respectively; and the coefficient of variation of R wave-to-R wave (RR) interval (cvRR), component coefficient of variation of LF (ccvLF), and component coefficient of variation of HF (ccvHF), reflecting fluctuation of RR interval, LF, and HF, respectively.

**Results:**

Higher J-OSDI scores were associated with higher HF, ccvHF, and subjective stress in sBUTDE, showing a significant correlation (r = 0.53, *P* < 0.01; r = 0.55, *P* = 0.01; and r = −0.66, *P* = 0.01); no correlations were observed between the J-OSDI score and autonomic parameters and stress in ADDE.

**Conclusions:**

DE symptoms were significantly associated with the magnitude and fluctuation of parasympathetic activity in sBUTDE. Thus, among the autonomic parameters, parasympathetic activity is involved in the development of symptoms in sBUTDE, whereas the involvement of the autonomic nervous system may be minimal in ADDE.

The number of individuals with dry eye (DE) is increasing rapidly due to lifestyle changes associated with the recent increase in the use of visual display terminals (VDTs) and the aging of the population.^1^^,^^2^ In particular, the proportion of individuals with short tear break-up time (BUT) DE (sBUTDE), which can be attributed to tear film instability, is high.^3^ Because no reduction in tear production and no staining of the ocular surface occurs in sBUTDE, the mechanism of symptom onset remains unknown. Neurological disorders may be involved in some cases of sBUTDE that are refractory to conventional DE treatment.^4^^,^^5^

Electrophysiological studies have clarified that changes in the responsiveness of transient receptor potential vanilloid-1 (TRPV1) and transient receptor potential melastatin-8 (TRPM8) channels present at the corneal nerve endings contribute significantly to the etiology of DE.^6^^–^^8^ However, many studies have used tear-deficient DE animal models with the lacrimal glands surgically removed.^6^^–^^8^ The response of TRPV1 and TRPM8 in sBUTDE, which shows no destruction of the lacrimal gland but accumulated enlarged secretory vesicles,^9^ has not been clarified. We propose that aqueous tear-deficient DE (ADDE), where inflammation destroys the lacrimal glands, and sBUTDE, where tear secretion remains unaffected, should be considered as separate entities.

We previously reported that emotional sensations, not somatosensory abnormalities, are involved in the neural pathways of perception in sBUTDE.^10^ Pleasant and unpleasant emotions organize the activity of the autonomic nervous system. We examined the autonomic nerve activity in sBUTDE and found that sBUTDE showed a large fluctuation in the autonomic nervous balance and that this fluctuation was related to the intensity of DE symptoms.^11^ Therefore, we speculated that higher brain function sensitized by DE status is involved in the development of symptoms and destabilization of the autonomic nervous balance in sBUTDE. In this study, we reversed these results in patients with symptomatically refractory sBUTDE and compared them with those of patients with ADDE, focusing on sympathetic and/or parasympathetic nerve activities.

## Materials and Methods

The study protocol was reviewed and approved by the Ethics Committee of the Institutional Review Board of Ito Clinic, Saitama, Japan. All procedures were performed in accordance with the ethical standards of the responsible committee on human experimentation (institutional and national) and the Helsinki Declaration of 1964, as revised in 2013. Informed consent was obtained from all participants. This study was registered with the University Hospital Medical Information Network (Registry ID: UMIN000047525).

### Participants

This prospective, cross-sectional, comparative clinical study included 25 eyes of 25 patients with sBUTDE (mean age = 57.4 ± 11.2 years, range = 30–75 years) and 24 eyes of 24 patients with ADDE (mean age = 62.3 ± 10.7 years, range = 29–76 years) who are refractory to conventional treatments, such as eye drops, anti-inflammatory therapy, and/or lacrimal punctal occlusion (Supplementary S1). The sample size was determined based on a previous study.^11^ Among the 24 patients with ADDEs, 14 had Sjögren's syndrome. The sBUTDE was diagnosed based on the presence of DE symptoms, tear BUT ≤5 seconds, and negative keratoconjunctival damage (keratoconjunctival vital staining <3 points). The Schirmer test value was not considered for the diagnosis of sBUTDE, according to the 2016 Japanese DE diagnostic criteria.^12^ ADDE was diagnosed based on the presence of DE symptoms, keratoconjunctival vital staining ≥3 points, and Schirmer test value ≤5 mm. The exclusion criteria were as follows: ocular surgery and trauma within the last 12 months, permanent structural damage due to ocular trauma, receiving pressure-lowering agents for the treatment of glaucoma, current contact lens use, and systemic diseases including metabolic, cardiovascular, and pulmonary diseases that may affect heart rate, other than hypertension. In addition, smokers and those taking medications for colds and allergies to pollen were also excluded. The eye with the strongest symptoms in each patient was selected for evaluation. The right eye was selected if the symptoms were equal in both eyes.

### Dry Eye Questionnaire

We administered the Japanese version of the Ocular Surface Disease Index (J-OSDI).^13^ Based on the J-OSDI symptom severity classification, in which Inomata et al.^14^ defined a J-OSDI score of 33 or higher as severe symptoms, patients with sBUTDE and ADDE were classified into 2 groups according to the symptom intensity: less than 33 points (sBUTDE1 and ADDE1) and 33 points or more (sBUTDE2 and ADDE2) in J-OSDI.

### Stress Check Questionnaire

We administered the second part of the Brief Job Stress Questionnaire, which included questions concerning the participants’ health during the past month.^15^ The questions consisted of 6 scales of liveliness, frustration, fatigue, anxiety, depression, and physical complaints, yielding a total of 29 questions. The patients selected one of the four following responses, “almost never,” “sometimes,” “often,” and “always,” and the score was calculated using a conversion table. The higher the stress, the lower the score, and the lower the stress, the higher the score (6–30 points).

### Slit-Lamp Examinations

The patients underwent slit-lamp examinations for lid margin abnormalities, including vascular engorgement, replacement of the mucocutaneous junction, irregular lid margin, plugging of the meibomian gland orifices, and foaming. These clinical signs were assessed at the upper and lower eyelids. The clinical severity grading scores were determined as 0 to 3 points for vascular engorgement and replacement of the mucocutaneous junction, and 0 to 2 points for irregular lid margin, plugging of the meibomian gland orifices, and foaming.

We performed ocular surface examinations, including BUT measurement, keratoconjunctival vital staining, and the Schirmer test. BUT was measured after instilling 2 µL of preservative-free 1% sodium fluorescein into the conjunctival sac using a micropipette. Keratoconjunctival epithelial staining was evaluated after BUT measurement. The overall epithelial damage was scored on a scale of 0 to 9 points, as described previously.^16^ The Schirmer test was performed last during eye surface evaluations.

### Autonomic Nerve Activity Measurement

We performed autonomic nerve activity measurements using the Silmee Bar type Lite (TDK, Tokyo, Japan) at least 10 minutes after the Schirmer test to minimize the effect of the Schirmer test on autonomic nerve activity. This biosensor automatically calculates the heartbeat intervals, pulse wave intervals, and autonomic nerve activity by measuring and analyzing the electrocardiogram (ECG) pulse waves, acceleration, and skin temperature. We extracted the data regarding the heart rate, R wave-to-R wave (RR) interval, low-frequency (LF) and high-frequency (HF) components of heart rate variability, and LF/HF ratio as autonomic parameters to avoid excessive amounts of data.

The device was attached approximately 3 cm below the center of both clavicles, a position where the electrocardiographic potential could be measured close to the heart, and the influence of the movement of the upper arm and chest muscles are limited. A previous report on autonomic nerve activity associated with aging and repositioning demonstrated that autonomic nerve activity is affected in the sitting position (sympathetic nerve activity decreases with age) but not in the supine position.^17^ Therefore, we measured the autonomic nerve activity with natural blinking for 10 minutes in the supine position^18^^,^^19^ to minimize the influence of age. The temperature of the room was maintained at 23°C to 25°C with 60% to 70% humidity during the examinations.

Autonomic nerve function was assessed by frequency analysis of the cardiac beat movements (fluctuations in heart rate [RR] intervals) and quantification of sympathetic and parasympathetic activity.^20^^,^^21^ This technique partitions the total variance (“power”) of a continuous series of beats into its frequency components, typically identifying two main peaks: LF (0.04–0.15 Hz) and HF (0.15–0.4 Hz).^18^^,^^19^ The HF peak reflects the cardiac parasympathetic nerve activity, whereas LF has a dominant sympathetic component.^16^^,^^17^ Based on these assumptions, it was proposed that the LF/HF ratio can be used to quantify the degree of sympathovagal balance.^20^^–^^25^ Furthermore, Hayano et al. proposed fluctuation coefficients calculated from the measured values: the coefficient of variation of the RR interval (cvRR), coefficient of component variance of LF (ccvLF), and coefficient of component variance of HF (ccvHF).^23^ The values of cvRR, ccvLF, and ccvHF were calculated using the following formulas: cvRR = RRsd/RR × 100; ccvLF = √LF/average(RR) × 100; ccvHF = √HF/average(RR) × 100. The heart rate variability metrics obtained in this study were the heart rate interval (RR intervals), LF, HF, LF/HF ratio, cvRR, ccvLF, and ccvHF.

### Statistical Analyses

Data are presented as the mean *±* standard deviation, wherever applicable. The correlation between the OSDI score and the DE and autonomic nervous activity parameters was analyzed using Pearson's correlation analysis separately in the sBUTDE and ADDE groups. The parameters of autonomic nervous activity were compared among the 4 groups classified based on the intensity of symptoms using 1-way ANOVA. Further analysis was performed using Dunnett's test. An ANCOVA test was performed to adjust for age and sex.

All statistical analyses were performed using SPSS statistical software (version 17.0 J for Windows; IBM, Armonk, NY, USA). Statistical significance was set at *P* < 0.05.

## Results

### Demographics of the Study Population

The Table shows the demographic characteristics of the patients in the sBUTDE1 and ADDE1 groups with symptom intensity of less than 33 points in the J-OSDI score and the sBUTDE2 and ADDE2 groups with symptom intensity of ≥33 points in the J-OSDI score. No significant differences in the grading scores relating to lid abnormalities among the four groups were observed (*P* > 0.05). The vital staining score was significantly higher, and the Schirmer test value was significantly lower in the ADDE1 and ADDE2 groups than those in the sBUTDE2 group. The stress levels were significantly lower in the sBUTDE1, ADDE1, and ADDE2 groups than those in the sBUTDE2 group (*P* < 0.05).

**Table.** Demographics of the Study Population

	sBUTDE		ADDE	
	J-OSDI <33	J-OSDI ≥33	J-OSDI <33	J-OSDI ≥33
	*N* = 13	*N* = 12	*N* = 9 (ss:7)	*N* = 15 (ss:7)
**Age** **,** **y**	60.5 ± 9.9	54.3 ± 12.3	67.2 ± 5.1*	58.1 ± 12.8
**Age range**	41–75	30–74	60–76	29–73
**Male/female**	2/11	2/10	0/9	0/16
**Vascular engorgement (points)**				
U	1.2 ± 0.9	1.2 ± 0.4	0.9 ± 0.6	0.9 ± 0.7
L	1.2 ± 0.8	1.2 ± 0.4	1.0 ± 0.9	0.9 ± 0.7
**Replacement of MCJ (points)**				
U	0.2 ± 0.4	0.3 ± 0.5	0.3 ± 0.5	0.2 ± 0.4
L	0.2 ± 0.4	0.3 ± 0.5	0.3 ± 0.5	0.2 ± 0.4
**Irregular lid margin (points)**				
U	0.1 ± 0.3	0.2 ± 0.4	0.3 ± 0.5	0.1 ± 0.4
L	0.1 ± 0.3	0.1 ± 0.3	0.3 ± 0.5	0.1 ± 0.4
**Plugging (points)**				
U	1.3 ± 0.9	1.0 ± 0.6	0.7 ± 0.7	0.8 ± 0.6
L	1.2 ± 1.0	0.8 ± 0.5	0.7 ± 0.7	0.7 ± 0.5
**Foaming**				
U	0.0 ± 0.0	0.08 ± 0.29	0.0 ± 0.0	0.0 ± 0.0
L	0.0 ± 0.0	0.08 ± 0.29	0.0 ± 0.0	0.0 ± 0.0
**BUT (s)**	3.4 ± 1.7	3.1 ± 1.8	2.0 ± 1.2	3.3 ± 3.0
**VS score (points)**	0.8 ± 1.2	0.5 ± 1.2	3.6 ± 2.8*	3.6 ± 2.5*
**Schirmer value (mm)**	7.4 ± 4.9	7.2 ± 4.4	3.0 ± 4.3*	3.4 ± 2.8*
**J-OSDI (points)**	20.2 ± 6.0*	49.3 ± 17.1	22.8 ± 7.5*	48.7 ± 12.1
**Mental stress**	22.7 ± 5.4*	17.3 ± 5.4	21.0 ± 3.6*	20.1 ± 3.0*
**Lacrimal punctal occlusion (at either punctum/at both puncta)**	0/0	0/0	0/1	6/3

One-way ANOVA (Dunnett test): *P < 0.05.

MCJ, mucocutaneous junction; BUT, tear break-up time; VS, vital staining; J-OSDI, Japanese version of the Ocular Surface Disease Index Questionnaire; DE, dry eye; sBUTDE, short tear break-up time dry eye; ADDE, aqueous deficiency dry eye; SS, Sjögren syndrome; U, upper eye lid; L, lower eye lid.

### Autonomic Nerve Activity

**Figure 1.**
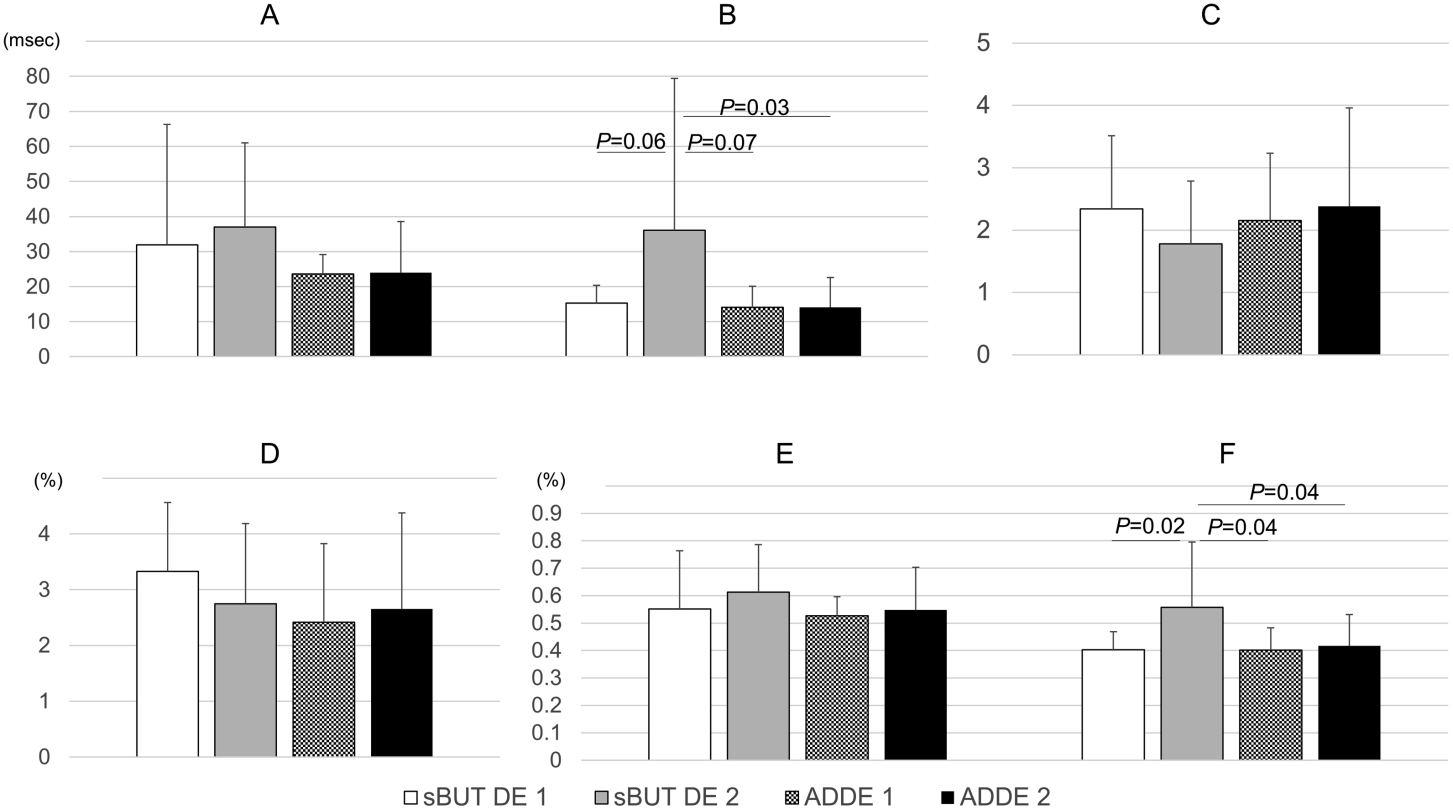
Autonomic nerve activity in the sBUTDE1, sBUTDE12, ADDE1, and ADDE2 groups. (**A**) LF; (**B**) HF; (**C**) LF/HF; (**D**) ccRR; (**E**) ccvLF; and (**F**) ccvHF. The HF and ccvHF values are significantly higher in the sBUTDE2 group than those in the other groups (*P* < 0.05; Figs. 2B, 2F). The LF and HF values in the ADDE1 and ADDE2 groups tend to be low (Figs. 2A, 2B), although the LF/HF ratio in the ADDE1 and ADDE2 groups is similar to that in the sBUTDE1 group (Fig. 2C). sBUTDE, short tear break-up time dry eye; ADDE, aqueous deficiency dry eye; LF, low frequency; HF, high frequency; ccRR, coefficient of variation of the RR interval; ccvLF, coefficient of component variance of low frequency; ccvHF, coefficient of component variance of high frequency.

**Figure 2.**
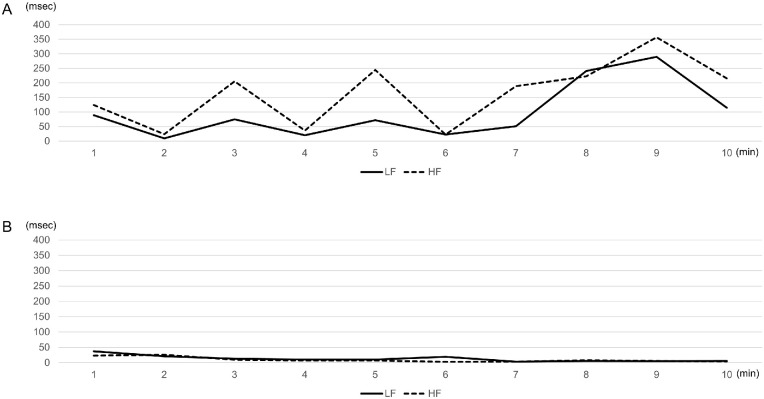
Typical cases with severe symptoms in the sBUTDE and ADDE groups. (**A**): A 40-year-old female patient with sBUTDE with a J-OSDI score of 79.5 points, BUT of 3 seconds, VS score of 0, and Schirmer value of 8 mm. (**B**) A 29-year-old female with Sjögren syndrome with a J-OSDI score of 62.5, BUT of 1 second, VS score of 7 points, and Schirmer value of 3 mm. J-OSDI, Japanese version of the Ocular Surface Disease Index Questionnaire; sBUTDE, short tear break-up time dry eye; ADDE, aqueous deficiency dry eye; BUT, tear break-up time; VS, visual analog scale.

The RR intervals showed no significant differences among the four groups (*P* > 0.05; 963.0 ± 81.6 in the sBUTDE1 group; 952.0 ± 122.1 in the sBUTDE2 group; 925.7 ± 141.3 in the ADDE1 group; and 850.5 ± 129.8 in the ADDE2 group). The HF and ccvHF values were significantly higher in the sBUTDE2 group than those in the other groups (*P* < 0.05; Fig. 1). The LF and HF values in the ADDE1 and ADDE2 groups tended to be low, although the LF/HF ratio in the ADDE1 and ADDE2 groups was similar to that in the sBUTDE1 group (see Fig. 1). Figure 2 shows the changes in LF and HF over time in typical cases with severe symptoms in the sBUTDE1 and ADDE2 groups. In the ANCOVA test, which examined the effects of age and sex on HF and ccvHF values, the fixed-factor HF value was *P* = 0.09 and the covariates age and sex were *P* = 0.12 and 0.21, respectively; the fixed-factor ccvHF value was *P* = 0.03, and the covariates age and sex were *P* = 0.46 and 0.06, respectively.

### Correlation Among DE Symptom Intensity, Stress, and Parameters of Autonomic Parameters

**Figure 3.**
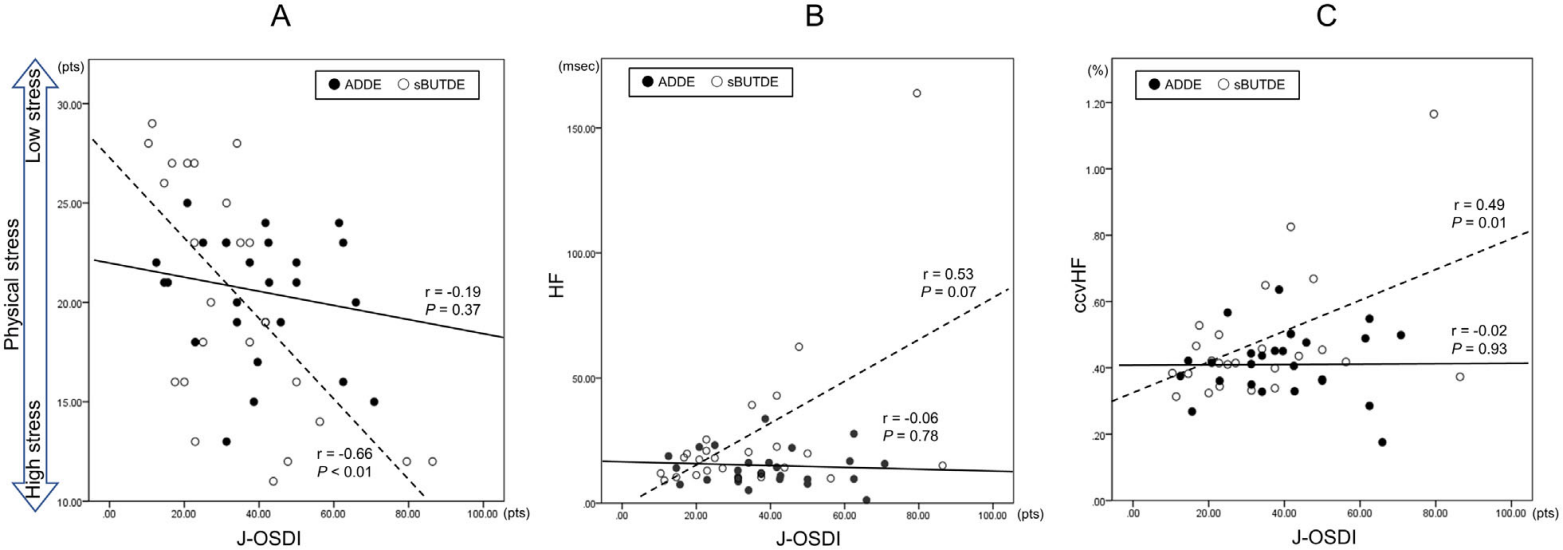
Correlation between J-OSDI, physical stress, HF, and ccvHF in the sBUTDE and ADDE groups. (**A**) Correlation between the OSDI scores and physical stress. (**B**) Correlation between the OSDI scores and HF. (**C**) Correlation between the OSDI scores and ccvHF. J-OSDI, Japanese version of the Ocular Surface Disease Index Questionnaire; HF, high frequency; ccv, coefficient of component variance; sBUTDE, short tear break-up time dry eye; ADDE, aqueous deficiency dry eye.

Significant correlations between the OSDI scores and stress (r = −0.657, *P* < 0.001), HF (r = 0.526, *P* = 0.007), and ccvHF (r = 0.489, *P* = 0.013) were observed in the sBUTDE group (Fig. 3^3^). No significant correlations between the OSDI scores and any of the autonomic parameters were observed in the ADDE group.

## Discussion

This study clarified the association between the intensity of DE symptoms and autonomic activity: there was a relationship between DE symptom intensity and parasympathetic activity in sBUTDE, whereas sympathetic and parasympathetic activity was low and there was minimal relationship between DE symptom intensity and autonomic activity in ADDE. In addition, stress was high in sBUTDE but not in ADDE. This suggests that the difference in autonomic nerve activity between sBUTDE and ADDE is related to the presence or absence of stress. In other words, there may or may not be a strong relationship between DE symptoms and stress, the former being sBUTDE and the latter being ADDE. These results were similar to those of our previous study.^11^ Of note, parasympathetic, but not sympathetic, activity plays an important role in the development of DE symptoms in sBUTDE. The autonomic nervous system plays a major role in the stress responses of the emotional pathways.^26^^,^^27^ The stronger the symptoms, the higher the stress, HF, and ccvHF in sBUTDE, suggesting that the emotional system controlling the motivational and emotional aspects is involved in inducing symptoms in sBUTDE. Psychogenic factors (emotional sensations) are related to pathogenesis in several diseases. Sympathetic nerve overactivation, heart rate, and itching increase when placed in a stressful environment or a state of high anxiety in atopic dermatitis.^28^ In contrast, an extreme drop in heart rate is observed at rest, and the autonomic nerves are modulated.^29^ Autonomic dysfunction, including low parasympathetic activity, has also been observed in bronchial asthma.^30^ Music therapy is effective in strengthening the parasympathetic nerve activity, reducing anxiety, and stabilizing breathing.^31^ Abnormal autonomic nervous activity is also observed in duodenal ulcers,^32^ musculoskeletal pain,^33^ and lower back pain,^34^ and mental and physical factors are considered to be involved in the onset of symptoms in these diseases. Similarly, sBUTDE may be a disease in which abnormal changes in the autonomic nerve system and psychosomatic factors are strongly involved in the induction of symptoms.

We initially assumed that DE states sensitize higher brain functions in sBUTDE, resulting in the development of symptoms and fluctuations of autonomic activity. However, despite the existence of an association between DE symptoms and stress, it seems contradictory that parasympathetic rather than sympathetic activity is involved in the development of DE symptoms. This may be related to the peculiarity of the eyes. The parasympathetic system plays a dominant role in the autonomic regulation of the eyes. For instance, the regulation of tear secretion requires parasympathetic activity.^35^^–^^37^ The pupillary and accommodative responses to visual stimuli also vary. VDT work, which causes DE and requires an accommodative response in near vision work, may put an unstable load on the parasympathetic activity. Thus, eye-induced stress may affect the parasympathetic activity.

On the other hand, instead of the DE state causing changes in autonomic nervous system activity, changes in autonomic nervous activity may cause the DE state. In other words, cause and effect may be reversed. Hyperactivity of parasympathetic nerves causes abnormalities in lipid secretion from the meibomian glands^38^^,^^39^ and mucin secretion from goblet cells^40^^,^^41^ under its control, possibly leading to qualitative abnormalities in the tears and developing DE with reduced tear stability. As a result, sympathetic activity may be masked by large fluctuations in parasympathetic activity, and sympathetic nerve activity may appear to be low. In fact, our results show a trend toward both higher HF and LF, which reflects sympathetic nerve activity, in sBUTDE compared to ADDE. Thus, the essential etiology of sBUTDE may be abnormalities in the autonomic nerve system. However, there was no difference in eyelid findings between DE groups in this study, suggesting that it was unlikely that lipid-secreting ability of meibomian glands by autonomic nerves affects tear quality. Further studies should investigate the parasympathetic nerve-related factors that cause a decrease in tear stability.

A different mechanism may be involved in the development of symptoms in ADDE compared with sBUTDE, because the autonomic nerve activity in ADDE was low and there was no association with symptom intensity. We speculated that the somatosensory system may be involved in the development of ADDE symptoms. Several studies using tear-reducing DE model animals with surgically removed lacrimal glands have shown that the high-threshold receptors of TRPM8 are hyperexcited by cold stimulation.^6^^–^^8^ This hyper-responsiveness of cold receptors may be transmitted to the somatosensory area and recognized as a symptom. In addition, the increase in inflammatory cytokines due to DE increases the sensitivity to pain and may induce DE symptoms.^42^^,^^43^ This speculation may be acceptable even from the results that DE parameters, such as BUT value, keratoconjunctival injury, and Schirmer test values, are not related to symptom intensity. In ADDE, the decrease in autonomic nerve activity leads to decreased lacrimal secretion, and further deterioration of the ocular surface may form a vicious cycle.

Our study has a few limitations. We did not examine confounding factors that could affect autonomic function, such as menopause, diet, lifestyle, mental and psychological problems, or the presence of other systemic diseases. Autonomic nerve activity is also affected by age and body position,^22^ but we did not examine the relationship between autonomic nerve activity and body position. Although there were age differences in the four groups and there were more women than men, ANCOVA showed that age and sex had no effect on the results of autonomic nerve activity. In this study, autonomic nerve activity was measured under one condition, natural blinking, but we would like to conduct further studies to investigate autonomic nerve activity under various conditions.

In conclusion, autonomic nervous activity, especially parasympathetic activity, appears to be involved in the development of symptoms in sBUTDE. However, the involvement of the autonomic nervous system appears to be low in ADDE. Fluctuations in autonomic nerve activity might be involved in the development of symptoms in sBUTDE. Thus, in addition to peripheral approaches for DE treatments, suppressing autonomic nervous system hyperexcitability might lead to symptom relief.

## Supplementary Material

Supplement 1
