# Supplementary material for: Autonomic Nerve Activity Features According to Dry Eye Type
Source: Invest Ophthalmol Vis Sci. 2023 Jun 12;64(7):19. doi: 10.1167/iovs.64.7.19 (PMC10266552; doi:10.1167/iovs.64.7.19)
Supplement: Supplement 1 [file iovs-64-7-19_s001.pdf]

No	ATD1sBUT DE2	age	M1F2	ss1	R1L2	OSDI	stress	ıl occlusi	BUT	F	S	HR
1	1	68	2	1	1	15.6	21	0	3	2	0	59.059
2	2	46	2		1	47.7	12	0	1	0	10	50.996
3	1	63	2	1	1	14.6	21	0	1.7	3	0	67.506
4	2	63	1		2	31.3	25	0	2.7	0	4	63.468
5	2	61	2		1	20.8	27	0	4.7	0	10	60.654
6	2	61	2		1	86.4	12	0	1	0	5	58.422
7	2	58	2		1	20	16	0	2.7	2	2	58.229
8	2	63	2		2	10.4	28	0	3	1	8	66.897
9	2	55	2		1	43.8	11	0	1.3	3	3	69.89
10	2	68	2		1	22.7	27	0	3	1	14	62.306
11	2	72	2		1	14.6	26	0	2	0	6	71.538
12	2	68	1		1	37.5	23	0	4	0	2	63.189
13	2	65	2		1	16.7	27	0	1	4	2	65.649
14	2	56	2		1	41.7	19	0	2.7	0	9	63.609
15	2	60	2		1	25	18	0	3.7	0	15	58.287
16	1	50	2	1	2	45.8	19	0	1.3	3	6	61.263
17	2	44	2		1	50	16	0	3.3	0	10	61.556
18	2	63	1		2	11.4	29	0	5.3	1	4	62.528
19	2	57	1		1	37.5	18	0	6.7	0	7	69.33
20	2	42	2		1	17.5	16	0	3.7	2	4	71.83
21	2	58	2		1	56.25	14	0	1	3	5	79.893
22	2	56	2		1	22.9	13	0	2	0	13	57.391
23	1	72	2	1	1	12.5	22	0	1.7	2	2	52.191
24	2	75	2		1	22.7	23	0	7.3	0	12	56.209
25	2	41	2		2	27.1	20	0	2.3	0	2	66.931
26	2	30	2		2	35	23	0	4.3	0	18	62.489
27	2	40	2		1	79.5	12	0	3	0	8	64.951
28	1	29	2	1	1	62.5	16	1	1	7	3	55.388
29	1	71	2	1	1	42.5	23	1	1	3	0	78.594
30	1	67	2	1	1	70.8	15	2	2	6	1	75.979
31	1	43	2		2	42.7	21	1	8	0	6	59.906
32	1	62	2	1	1	31.3	13	0	1	6	0	71.412
33	1	60	2		1	25	23	0	1	9	0	71.127
34	1	65	2		1	34.1	20	1	8	2	6	65.531
35	1	57	2		1	38.6	15	0	5	2	0	65.885
36	1	66	2		2	20.8	25	0	2	2	1	52.728
37	1	66	2		2	34.1	19	0	3	2	2	86.756

38	1	69	2	1	1	31.23	23	0	1	2	4	76.752
39	1	68	2		1	41.7	24	0	1	8	7	80.463
40	1	73	2		1	37.5	22	0	1	6	2	79.645
41	1	69		1	2	22.9	18	2	4.7	0	8	71.286
42	1	61		1	2	39.6	17	2	9	1	2	68.387
43	1	47			1	50	22	1	2	2	3	70.95
44	1	63		1	1	50	21	1	0	5	5	78.184
45	1	70		1	1	65.9	20	2	5.3	0	9	96.886
46	1	76			1	31.2	23	0	2	6	12	74.1
47	1	59		1	1	62.5	23	0	1.3	5	0	62.684
48	2	74	2		1	41.7	19	0	3.7	0	6	78.658
49	2	60	2		1	34.1	28	0	5	0	3	61.216

HRsd	RR	LF	HF	LFHF	CVRR	CCVLF	CCVHF
0.9286575	1016.5	28.907677	7.4393002	4.6459114	1.5680394	0.5289312	0.2683235
1.2650897	1182.4	24.150866	62.494633	0.398081	2.0942133	0.4156253	0.6685855
1.748224	889.4	18.544118	14.036657	1.4165443	2.5983137	0.484179	0.421245
1.6781669	946.3	16.234249	9.8536763	2.225946	2.533281	0.425782	0.331719
1.6304622	990.4	19.992451	17.420445	1.295935	2.6274447	0.4514632	0.4214237
1.8311035	1038.1	52.452238	15.007705	3.4806968	2.1609062	0.6976584	0.3731796
2.0883369	1031.7	14.817754	11.186299	1.402115	3.5260058	0.3731108	0.3241827
1.4073639	898.2	24.078021	11.901201	2.255335	2.186606	0.5463077	0.3840806
2.4582044	865.3	26.75857	14.214578	2.5519158	3.2841462	0.5978122	0.4357128
3.3863077	1008.7	145.19807	25.434268	4.9822157	2.3497657	1.1945887	0.4999742
3.8039255	842.33333	26.599986	10.37325	3.2607354	5.157399	0.6122894	0.3823609
0.5290182	950	17.589991	10.344853	1.8807302	0.7985829	0.4414781	0.3385623
1.5558486	915.2	21.641255	18.187944	1.6760258	2.2715129	0.508306	0.4659891
1.8981814	944.6	16.519276	22.534654	0.789746	2.9283766	0.4302764	0.502548
3.2774351	1034.3333	25.028951	18.002103	1.882855	5.0026972	0.483683	0.4102051
2.6659532	987.1	42.805572	22.116632	2.049015	4.3357152	0.6628099	0.4764292
1.6465267	980.22222	45.527664	19.829588	3.3847059	2.4752577	0.6883561	0.4542891
1.6202105	961.1	26.258459	9.0810056	4.3724509	2.5184593	0.5331704	0.3135439
2.4773021	866.88889	18.802804	11.973558	1.8341624	3.3692975	0.5002048	0.3991611
4.1655807	841.2	33.01918	19.726836	2.0198736	5.855742	0.6830994	0.5279946
1.5748903	751.4	13.262317	9.8538496	1.4567013	1.991042	0.4846615	0.4177647
2.0476357	1047.1	17.773682	12.971895	1.914297	3.505047	0.4026248	0.3439644
1.166483	1154.3	32.404744	18.764751	1.8541366	1.7389213	0.4931575	0.3752777
2.324501	1103.9	20.205044	20.900968	1.0964211	2.9648846	0.4071929	0.414146
1.9414647	899	24.026578	13.90109	2.0546305	2.7722025	0.5452382	0.4147293
1.8055007	965	38.190354	39.22665	1.1158822	2.4692319	0.6403974	0.6490278
4.0604941	1098.4	98.256395	163.89408	0.6009761	2.9038929	0.9024432	1.1655237
0.8835714	1089.2	13.093295	9.6688908	1.6832596	0.4737121	0.3322132	0.2854833
1.2376402	763.8	17.071792	9.5996984	1.0976533	1.5781074	0.5409535	0.4056478
5.2318126	794.1	50.810192	15.697268	2.2685884	6.9631107	0.8976359	0.4989268
1.3709058	1003	25.158807	10.915039	3.5582436	2.1619749	0.5000853	0.329391
1.3514262	842.22222	19.689784	8.6746666	2.3678889	1.89953	0.5268582	0.3497033
1.7625033	849.22222	29.845512	23.145715	1.6706848	1.6229007	0.6433068	0.5665183
2.574288	919	30.562015	16.109493	2.0290676	3.919316	0.6015552	0.4367425
1.0208555	912.9	28.1402	33.708691	0.8894236	1.5869382	0.581086	0.6359863
1.0821354	1138.8	22.504472	22.386572	1.089098	2.0155508	0.416569	0.4154764
0.9464388	691.3	13.132787	5.135456	2.6060719	1.0932915	0.5242179	0.3278106

0.9778367	782.3	22.871535	10.354457	2.236376	1.2740852	0.611328	0.41133
2.1719178	754.8	14.429248	14.359479	1.3647387	5.1409353	0.5032572	0.502039
1.2358142	754.5	7.8465415	11.588243	0.738021	1.5993297	0.3712615	0.4511797
2.3281147	844.9	21.60997	9.2948804	2.7870993	3.2203649	0.5502015	0.3608416
2.5672514	892.4	50.1865	16.158893	5.3199396	2.6702175	0.7938417	0.4504496
1.665689	846.3	26.494168	9.5334	3.1284083	2.3691616	0.6082061	0.3648376
1.4363729	768.1	12.041495	7.702047	1.5484558	1.7834583	0.4517753	0.3613144
2.2980212	619.7	4.6517623	1.1817174	6.1333185	2.2770804	0.3480385	0.1754185
4.5023792	813.4	15.869921	13.003093	1.3009356	5.8186757	0.4897599	0.4433219
1.1755405	960.7	23.489557	27.730192	1.3549097	1.8743083	0.5044866	0.5481364
3.9891922	794.2	54.79929	42.972749	1.5801114	6.7173987	0.9320894	0.8254042
1.4106372	987.4	37.813649	20.392689	2.2836621	1.7947437	0.622775	0.4573452
